# The relationship between inhibition of automatic imitation and personal cognitive styles

**DOI:** 10.1186/s40101-018-0184-8

**Published:** 2018-10-29

**Authors:** Yuki Nishimura, Yuki Ikeda, Shigekazu Higuchi

**Affiliations:** 1 Graduate School of Integrated Frontier Sciences, Kyushu University, 4-9-1 Shiobaru, Minami-ku, Fukuoka, 8158540 Japan; 2 Research Fellow of the Japan Society for the Promotion of Science, 4-9-1 Shiobaru, Minami-ku, Fukuoka, 8158540 Japan; 3 Faculty of Design, Kyushu University, 4-9-1 Shiobaru, Minami-ku, Fukuoka, 8158540 Japan

**Keywords:** Imitation inhibition, Personal cognitive traits, Mirror neuron system, Mu rhythm suppression

## Abstract

**Background:**

Previous studies have demonstrated the importance of the inhibition of automatic imitation in social interactions. Additionally, cognitive traits are known to vary among individuals. According to the empathizing-systemizing (E-S) model, personality can be quantified by empathizing and systemizing drives in causal cognition. Since inhibition of automatic imitation is strongly related to social cognition, the level of inhibition may be explained by personal cognitive traits. Thus, the current study tested whether cognitive traits, measured based on the E-S model, correlated with levels of automatic imitation inhibition.

**Methods:**

The empathizing-systemizing quotient (EQ-SQ) questionnaire was used to assess cognitive traits. Behavioral and electroencephalogram data were acquired during the imitation inhibition task. In addition to reaction time, based on signal detection theory, task sensitivity and response bias were calculated from reaction data. As a physiological measure of automatic imitation, mu rhythm power suppression was calculated from electroencephalogram data. Congruency effects for reaction time and electroencephalogram measures were calculated by subtracting congruent trials from incongruent trails.

**Results:**

Correlation analyses between cognitive traits and task measures were conducted. There was a negative correlation found between EQ score and the behavioral index reflecting task performance. Moreover, a negative correlation was found between SQ score and the congruency effect on mu suppression.

**Conclusions:**

Participants with higher EQ scored relatively lower in inhibiting their responses. Conversely, high SQ participants showed successful inhibition of mu suppression. The imitative tendency may disturb the inhibition of response. The correlation between SQ and mu index suggests the involvement of domain-general information processing on imitation inhibition; however, further research is required to determine this. Since different correlations were found for behavioral and physiological measures, these measures may reflect different steps of information processing for successful task execution. Through correlational analysis, a possible relation was identified between the inhibiting process of automatic imitation and personal cognitive styles on social interactions.

## Background

Throughout human evolution, cooperation (and resultant society building) has been selected for ultimately increasing the species’ chances of survival. As a social animal, it has been suggested that humans possess enhanced social cognitive abilities compared to any other creature [1]. Of these abilities, empathy is likely one of the most important, not only for the creation of societies, but also for the acquisition of motor skills [2–4]. Automatic imitation is one of the core functions of empathy. The neural basis of imitation is the mirror neuron system (MNS) [5, 6]. The MNS is a brain system first found in monkeys, which activates during both the performance and observation of a body action [5, 7–9]. A number of brain imaging and single-cell recoding studies have also demonstrated the existence of an MNS in humans [10–13]. It is thought that automatic imitation is one kind of stimulus-response compatibility that is acquired during development [14–16]. Several experiments and meta-analyses have shown that suppression of electroencephalogram (EEG) sensory-motor mu wave possibly reflects MNS activity, as mu wave power is suppressed by both the execution and observation of an action [17–21]. Especially, hand movements have a pronounced effect on imitative brain response measured by mu suppression [18, 22]. Moreover, spontaneous recording of fMRI and EEG have shown that mu wave reduction is one reliable measure of MNS activity [17, 21, 23].

Although automatic imitation, supported by the MNS, is a core function of social interaction, imitation is obviously not always the most appropriate reaction in daily life. Inhibition of imitative behavior is important for the facilitation of smooth complementary and joint actions. Therefore, in some cases, mirroring may be counterproductive [24–27]. For example, while handing a cup from one person to another, or while playing catch, the receiver should inhibit his/her imitative motor action and prepare an appropriate response. Furthermore, not only the actual imitation but also the in-brain imitation must be inhibited in the same manner for successful interaction. There is an ongoing debate as to which brain network is responsible for controlling automatic imitation. Initially, “social brain” regions, related to theory of mind, were found to be active during an imitation inhibition task. Brass et al. investigated the inhibitory system of imitative response tendencies and reported several brain regions involved in inhibition, including the temporal parietal junction, dorsolateral prefrontal cortex, right frontopolar cortex, right anterior parietal cortex, and precuneus [25, 28]. More recently, a domain-general brain network related to broad reaction-inhibition has also been implicated in imitation inhibition [29]. The imitation-inhibition task is designed to evaluate the level of inhibition of imitative tendencies [28]. It has been adopted in a number of studies to test the conditional or individual ability to inhibit the imitative response [25, 26, 28–34].

According to Decety and Svetlova, empathy is a complex construct consisting of both emotional and cognitive elements [2]. Furthermore, there has been an attempt to quantify human personality from causal cognition. Baron-Cohen developed the empathizing-systemizing (E-S) model to explain individual cognitive traits from two psychological “drives” [35]. Empathizing is a drive that identifies other’s emotions or thoughts and causes an individual to react with an appropriate emotion by analyzing psychological causal relations. By contrast, systemizing is a drive that analyzes factors of systems and derives basic patterns, which defy the function of the system. The term “system” here covers all processes, from physical law to social phenomenon, which have any kind of law-like nature [35, 36]. The Empathizing-Systemizing Quotient (EQ-SQ) questionnaire was developed to measure these constructs [37, 38].

Variation between individuals is known to exist in imitation-inhibition task performance and EQ-SQ score, both of which are thought to reflect social interactive skills. However, no study has aimed to address the relationship between an individual’s cognitive style (as assessed via the EQ-SQ questionnaire) and adaptive imitation-inhibition performance, despite both being closely related to social cognition and interaction in daily life and both possibly originating from higher order frontal brain functions. By investigating the relationship between personal cognitive traits assessed by the self-completion questionnaire and the performance of inhibition of automatic imitation, we aimed to deepen understanding of the involvement of the inhibiting process of automatic imitation in forming in individual’s traits related to social interaction.

To accomplish this, participants were asked to respond by lifting up either the index or middle finger, according to the task indicator and hand movement stimuli presented on the screen. Reaction time (RT), task sensitivity, and response bias were collected as behavioral measures and event-related mu wave desynchronization (ERD) of EEG was measured to determine imitating MNS activity and its modulation. We used indices from signal detection theory (SDT) instead of the classical proportion of correct answers. By using SDT measures, participant’s task performance (*d*’) is estimated independently from their response bias (*C*) [39–41]. We hypothesized that participants with a high EQ would show better performance on the task and successful inhibition of imitative brain activity.

## Methods

### Participants

Twenty-six young adults participated in the study (15 male, 11 female; mean age 23.2 ± 1.25 years). All participants were right-handed, as confirmed by an Edinburgh Handedness inventory (min–max 70–100; median 84.12). Participants were naïve as to the purpose of the experiment and were informed before the study that their privacy would be secured. Written informed consent was provided by the participants prior to the commencement of the experiment, and participants were debriefed following experiment completion.

The procedure of the study was approved by the Ethics Committee of Kyushu University. The study was conducted according to the principles of the Declaration of Helsinki.

### Equipment

The study was conducted in an acoustically and electrically sealed room located in Kyushu University. A 64-channnel EEG was recorded using an EEG amplifier (Net Amps 200, EGI) with sensor-net (HCGSN-64, EGI). Electrode impedance was maintained to remain under 50 kΩ as suggested by the manufacturer. EEG data were filtered in real time by the amplifier hardware, with the filter set to 0.01 Hz high-pass and 200 Hz low-pass, and digitized at 500 Hz. Stimuli were delivered with Presentation Ver. 20.0 (NBS Inc.) and an LCD display (E2351VR-BN, LG Electronics) refreshing at 60 Hz. Participants reaction (finger lifting) was acquired by capacitance sensor (AD00019, Bit Trade One, LTD.) connected to the Presentation software.

### Imitation-inhibition task

**Fig. 1:**
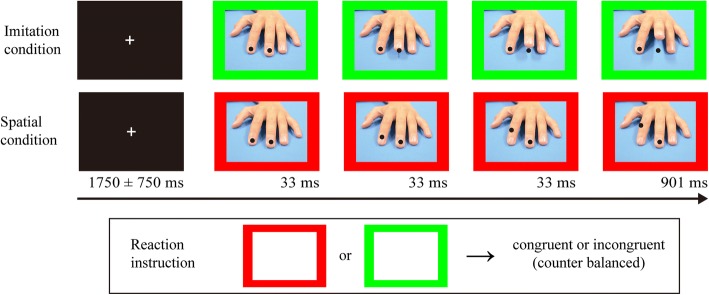
Example of the stimuli for imitation and spatial conditions. Instruction (congruent or incongruent) assignment to the colors (red or green) was counter balanced among participants. Presentation order of fingers (index or middle finger) and colors (red or green) in each condition was randomized. For example, if red was assigned to “congruent” and green was assigned to “incongruent,” lifting index finger is the correct response in both cases showed in the figure

The task utilized in the current study was based on a task developed and used in previous research [42]. Participants were required to respond as soon as possible according to simultaneously displayed finger movement and instructions (congruent or incongruent; Fig. 1). The presented stimuli consisted of a short movie of a hand quickly lifting either an index or middle finger, and a surrounding colored frame (red or green) indicating a response by the congruent or incongruent finger. The assignment of colors to the tasks (congruent or incongruent) was counter balanced among the participants. For example, if the index finger was lifted in the stimuli and the colored frame indicated an incongruent response, the correct response for the participant was lifting their middle finger.

In addition to the imitation condition described above, a spatial condition, in which one of two black-dots moved upwards, was tested to take the spatial compatibility aspect into account. The order of the conditions was counter balanced.

The task was divided into three blocks, including a randomization of trials, in which half were congruent and the other half were incongruent. This was done so that participants could not predict the next task instruction (total 300 trials per condition; 75 trials each for congruent index finger, congruent-middle finger, incongruent index finger, and incongruent middle finger).

### Behavioral measurements and analysis

Reaction time (RT) was calculated for each task instruction (congruent or incongruent) of each condition (imitation or spatial). In order to highlight the congruency-effect, ΔRT was calculated by subtracting the mean RT of congruent trials from the mean RT of incongruent trials for each condition.

Based on signal detection theory (SDT), task sensitivity index (*d’*), and response bias (*C*) were calculated as performance indices. SDT assumes that, in an uncertain condition, participants are active decision makers who make difficult perceptual judgements. The SDT index, rather than the percent of correct responses, provides effective solutions to differentiate participants’ response tendencies (bias) from the ability to detect and discriminate information (task sensitivity) [39–41]. We calculated sensitivity and bias by categorizing congruent success, congruent failure, incongruent failure, and incongruent success as hit, miss, false alarm, and correct rejection, respectively. A higher *d’* indicates higher task performance. The response bias index (*C*) is zero when there is no bias at all. This variable would take a positive value when responses are biased toward incongruent responses, and vice versa. Formulas for *d’* and *C* are the following:

$$ {d}^{\prime }={Z}_{\mathrm{hit}}-{Z}_{\mathrm{false}\ \mathrm{alarm}} $$

$$ C=-0.5\times \left[{Z}_{\mathrm{hit}}+{Z}_{\mathrm{false}\ \mathrm{alarm}}\right] $$

where, *Z*_hit_ is the *z*-transformed hit rate [hit count/(hit count + miss count)] and *Z*_false alarm_ is the *z* transformed false alarm rate [false alarm count/(correct rejection count + false alarm count)].

### EEG measurements and analysis

As an index of automatic imitation, mu wave event-related desynchronization (ERD) measured around central sulcus was acquired. In addition to mu ERD, alpha ERD possibly reflecting the attention level was calculated from the occipital sites, in order to take alpha wave contamination to the mu ERD into account. All EEG preprocessing and analysis was carried out using EEGLAB 14.1.1b [43], which is an open source toolbox of MATLAB (MathWorks Inc.). Raw EEG data, following manual rejection of bad channels, were filtered by FIR band-pass filter (0.5–40 Hz; transition band width 1 Hz) and epoched according to stimuli onset. Furthermore, bad epochs found in the data were automatically rejected according to joint probability of the data (both single-channel and all-channel threshold were set to 3 S.D). Following this, all data were re-referenced to the average. While data were re-referenced, outer electrodes (e.g., facial electrodes) were excluded from calculations (channels 23, 55, and 61 ~ 64). Two participants were excluded from further analysis due to an insufficient number of epochs. Preprocessed data were then sent to infomax independent component analysis (ICA). Independent components representing eye-blinks or eye-movement were manually rejected based on the topographical map, frequency spectrum, and activation synchrony with electrooculography (EOG).

**Fig. 2:**
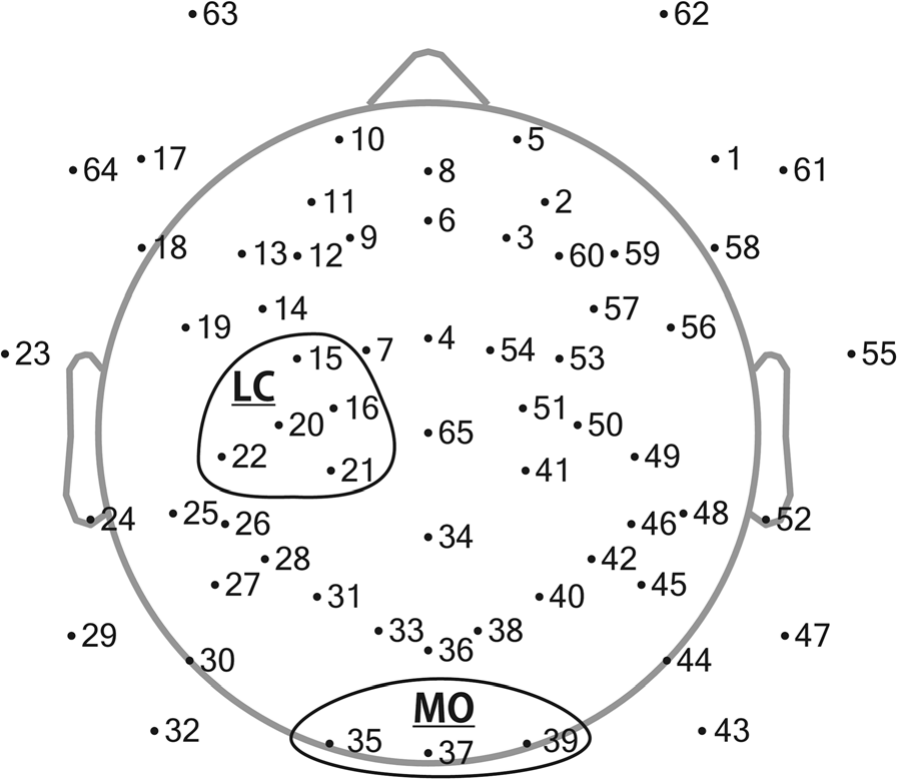
Channel location of the EEG sensor net. Each dot represents 1 of 65 channels. Channels included in the ROIs are circled

ERD was calculated by EEGLAB’s time-frequency analysis function. First, event-related spectrum perturbations were calculated by wavelet analysis, starting with 2 cycles and increasing by 0.5 cycles toward higher frequencies. Second, mu wave (8–13 Hz) power from 300 ms to 600 ms after the event onset was calculated for each participant in decibel, according to the baseline period, which was 200 ms to 0 ms prior to the event onset. Finally, ERDs from each channel were averaged across the regions of interest (ROIs) to improve the reliability of the data. The left central (LC) region, representing mu ERD, was covered by C3 and its neighboring channels (i.e., 15, 16, 20, 21, and 22). The mid occipital (MO) region, representing alpha ERD, was covered by Oz and its neighboring channels (i.e., 35, 37, and 39; Fig. 2). After calculating ERDs, a congruency effect for mu and alpha ERDs was calculated in the same manner as ΔRT.

### Statistical analyses

In order to test the relationship between the task performance and the EQ-SQ scores, Spearman’s rank correlation was calculated. A Bonferroni correction was used to adjust the *p* value for multiple tests. To highlight the effect of task and personal cognitive traits, an additional mixed-design analysis of variance (ANOVA) was conducted on data that showed significant correlations. The factors included in ANOVAs were high and low groups of EQ or SQ, and indicated tasks (congruent and incongruent). High EQ or SQ groups contained the upper 33% of participants and low EQ or SQ groups consisted of the lower 33% of participants. An independent *t* test was conducted to test the shift of response bias. All results are reported using an α of *p* < 0.05. All statistical tests were conducted using R version 3.5.1 [44].

## Results

### EQ-SQ score

**Fig. 3:**
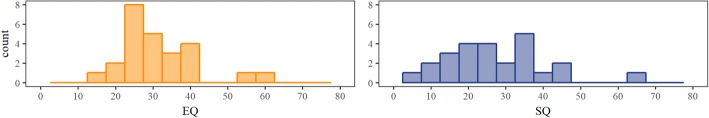
Histogram of EQ and SQ score distribution. Correlation analysis found no correlation between these scores (rho = 0.333, *n* = 25, *p* = 0.103)

EQ and SQ distribution of the participants are shown in Fig. 3. Both EQ and SQ varied widely (median = 29, 29; min = 7, 16; max = 58, 64; for EQ and SQ, respectively). No correlation was found between these two scores (rho = 0.333, *n* = 25, *p* = 0.103).

### Behavioral measures

**Fig. 4:**
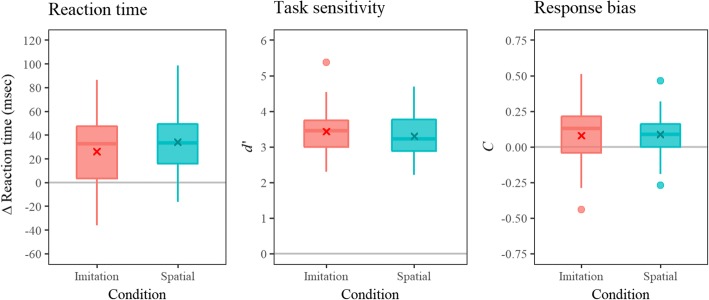
Distribution of ΔRT, task sensitivity (*d*’), and response bias (*C*) for each condition. ΔRT was calculated by subtracting the RT of congruent trial from incongruent trial in order to highlight the effect of imitative congruency. Higher task sensitivity scores indicate higher task performance. A zero value in the response bias indicates that there was no response bias observed. Since the distribution is slightly biased toward a positive value, it is indicated that the participant’s reactions were biased toward incongruent reactions, confirmed by a one-sample *t* test

Figure 4 shows the distribution of ΔRT, *d*’, and *C* for each condition. Generally, RT for the incongruent trials was slower than that of congruent trials, similar to results reported in earlier studies [26]. Although task sensitivity (*d*’) was generally high, it ranged from nearly 2 to 5. A one sample *t* test showed that response biases (*C*) were shifted toward a positive value, indicating that reactions were biased toward incongruent reactions in both the imitation and spatial conditions [*t*(24) = 24.582, *p* < 0.001; *t*(24) = 24.773, *p* < 0.001, respectively].

The results of a Spearman’s correlation test for the behavioral indices are shown in Table 1. In opposition to our hypothesis, negative correlations between EQ and ΔRT in the imitation condition and between EQ and *d*’ in the spatial condition were found, although the *p* values did not reach statistical significance after Bonferroni correction.

**Table 1:** Correlation results for behavioral measures

Condition	Index	EQ		SQ	
		rho	*P*_corrected_	rho	*P*_corrected_
Imitation	ΔRT	− 0.43	0.06†	0.07	1.00
	*d’*	− 0.20	0.68	− 0.25	0.45
	*C*	0.06	1.00	− 0.27	0.37
Spatial	ΔRT	− 0.29	0.31	0.04	1.00
	*d’*	− 0.43	0.06†	− 0.20	0.69
	*C*	0.27	0.40	− 0.37	0.13

Spearman’s rank correlation test

*n* = 24; Bonferroni corrected: † for *P*_corrected_ < 0.1

**Fig. 5:**
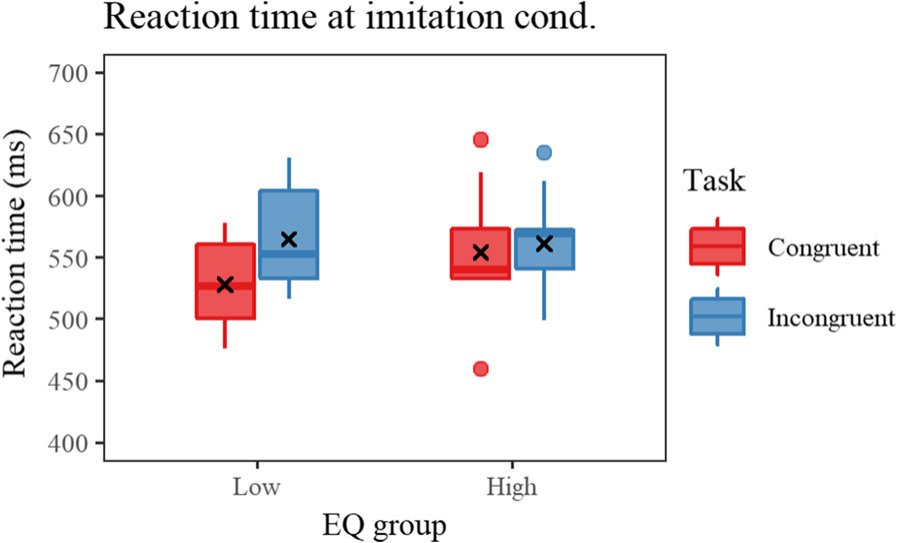
Reaction time at imitation condition for low and high EQ group. Red and blue boxes represent reaction time of congruent and incongruent trials, respectively. Shorter reaction time is interpreted as better performance. As a result of post hoc test following the ANOVA, a significant simple main effect of task was found in the low EQ group

A supplemental ANOVA on RT measured at imitation condition with task (congruent and incongruent) and EQ group (high and low) as factors revealed a significant main effect of task [*F*(1, 17) = 10.605, *p* = 0.005, η_p_^2^ = 0.384] indicating prolonged RT in incongruent trials, and an interaction of the two factors [*F*(1,17) = 4.785, *p* = 0.043, *η*_p_^2^ = 0.220]. Post hoc tests on the interaction showed a significant simple main effect of task for the low EQ group [*F*(1,9) = 14.680, *p* = 0.004, η_p_^2^ = 0.620] indicating shorter RT in the congruent trials. On the other hand, there were no RT differences in the high EQ group [*F*(1,8) = 0.586, *p* = 0.466, η_p_^2^ = 0.068]. As illustrated in Fig. 5, smaller ΔRT in participants with higher EQ originated from slower RT in the congruent trials. There were no significant correlations found between SQ and behavioral indices.

### Physiological measures

**Fig. 6:**
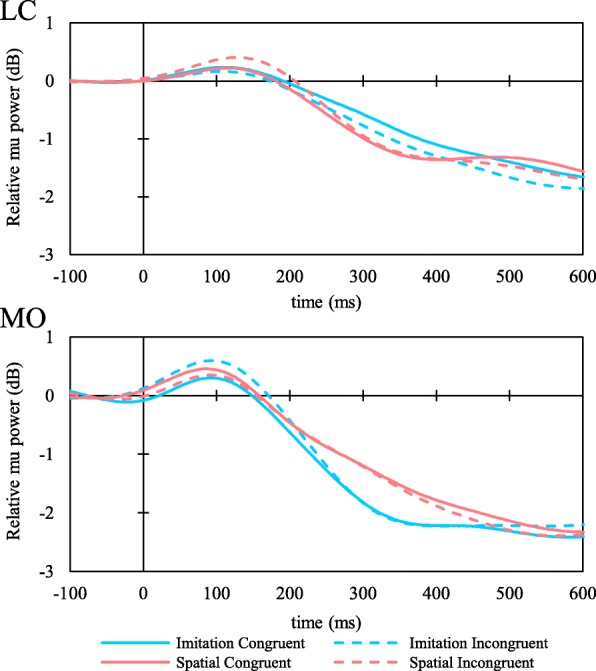
Grand average of mu and alpha ERDs measured at ROIs (LC and MO), respectively. Red lines represent imitation condition and blue lines represent spatial condition. Solid lines represent congruent trials and dotted lines represent incongruent trials for each condition

**Fig. 7:**
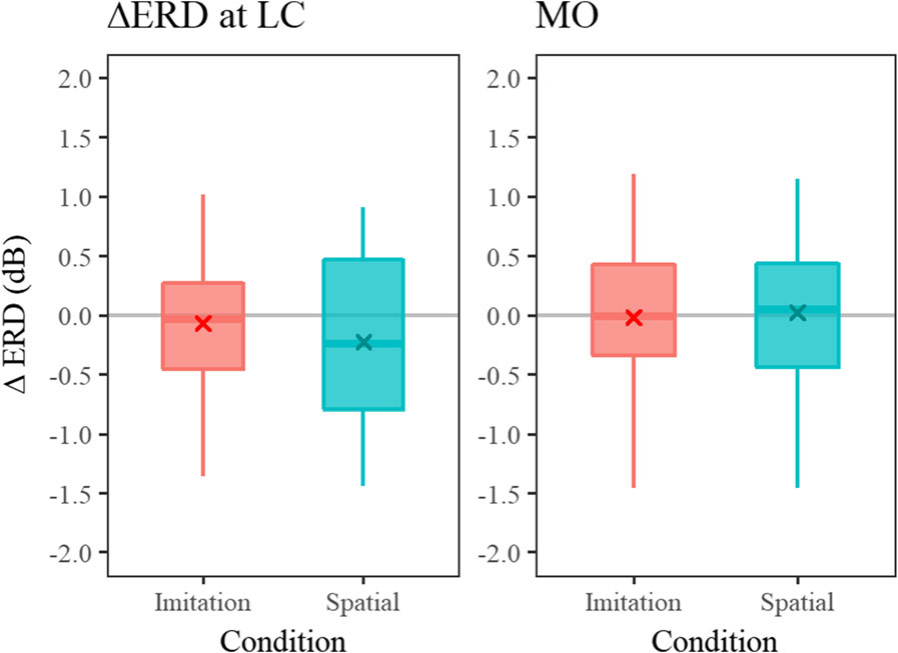
The observed congruency effect on mu and alpha ERD for each condition. Higher values of ΔERD indicate greater activity during the incongruent trials compared to the congruent trials and vice versa

Grand averaged mu and alpha ERD waveforms are shown in Fig. 6. The distribution of ΔERD measured over the LC (mu ERD) and MO (alpha ERD) are shown in Fig. 7. Higher Δmu ERD indicates a more successful inhibition of MNS activity during incongruent trials. Conversely, negative values of Δmu ERD indicate greater inhibition of MNS activity during congruent trials. All ΔERD are distributed from positive to negative values.

**Fig. 8:**
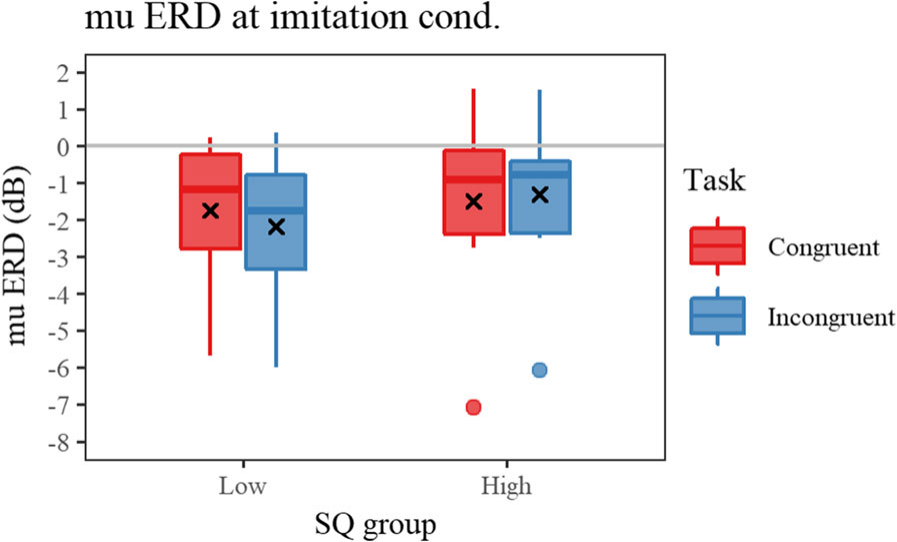
The mu ERD at imitation condition for low and high SQ groups. Red and blue box represents reaction time of congruent and incongruent trial, respectively. The greater ERD (more negative value) indicates greater sensory-motor activation including automatic imitation. The post hoc test following the ANOVA showed significant simple main effect of task for the low SQ group, indicating greater mu ERD at incongruent trials only for the low SQ group

The correlation analysis conducted on ΔERD revealed a positive correlation between SQ and Δmu ERD in the imitation condition (Table 1). An additional ANOVA on ERD measured at LC in the imitation condition with task (congruent and incongruent) and SQ-group (high and low) as factors was conducted. As a result, a significant interaction of the two factors was found [*F*(1,16) = 6.266, *p* = 0.024, η_p_^2^ = 0.281]. Post hoc analysis on the interaction showed a significant simple main effect of task for the low-SQ group indicating greater mu suppression in the incongruent trial [*F*(1,8) = 6.247, *p* = 0.037, η_p_^2^ = 0.439] (Fig. 8). No significant correlation was found between EQ and ΔERDs. In addition, there were no significant correlations found in the spatial condition (Table 2).

**Table 2:** Correlation results for EEG measures

Condition	ROI	EQ		SQ	
		rho	*P*_corrected_	rho	*P*_corrected_
Imitation	LC	0.22	0.58	0.46	0.04 *
	MO	− 0.16	0.88	− 0.02	1.00
Spatial	LC	− 0.20	0.69	− 0.11	1.00
	MO	− 0.12	1.00	− 0.01	1.00

Spearman’s rank correlation test. *ROI* region of interest; *LC* left central; *MO* mid occipital. *n* = 25; Bonferroni corrected: * for *P*_corrected_ < 0.05

## Discussion

In order to reveal the involvement of inhibition of imitation on formation of personal cognitive traits, we tested the relationship between personal cognitive traits, and performance on an imitation inhibition task, as measured by behavioral and physiological indices. In the imitation condition, a negative correlation was observed between EQ and ΔRT, although *p* values did not reach statistical significance following Bonferroni correction. Moreover, a positive correlation was observed between SQ and the congruency effect, as calculated from the mu ERD, which is an EEG index of MNS activity. These correlations were specific to the imitation condition, thus the corresponding correlations in the spatial condition were not significant. In the spatial condition, which was set to take the spatial compatibility effect into account, a trend of correlation between EQ and *d’*, which was specific to this spatial condition, was found. No correlation was found for ΔERD.

The ΔRT of the imitation condition showed a trend of negative correlations with EQ after the correction of multiple tests. A smaller ΔRT can be interpreted as a diminished congruency effect, which can further be interpreted as higher task performance. Nevertheless, in the current study, smaller ΔRTs originated from prolonged RTs in the congruent trials in high-EQ participants, suggesting that they took longer to respond in both congruent and incongruent trials. Therefore, higher empathic cognitive traits may lead to poorer imitation inhibition performance. From the related early research, it is known that the amount of unconscious imitation is influenced by the amount of rapport feeling [45, 46]. Moreover, one study, which primed participants either pro- or non-socially and compared the congruency effect, found that pro-socially primed adults showed a larger compatibility effect in an imitation task [47]. Later research also confirmed the effect of priming, especially in adults compared to adolescents (mean ages around 27 and 13 years, respectively) [48]. As such, higher empathic traits may disturb smooth selection (resulting in poorer reaction time) during the imitation inhibition task. Meanwhile, both empathic traits and higher imitation-inhibition ability are often associated with higher social ability and/or successful interactions [5, 9, 49]. Since the correlation found was negative, the current results suggest that a relationship exists between empathic cognitive traits and task performance but not in a direct manner. Further study is required to reveal the causal relationship related to this point.

The current study is the first to apply SDT to investigate the inhibition of automatic imitation. A number of earlier studies have used ΔRT to highlight the congruency effect of behavioral measures [26, 28, 33]. Compared to reaction time, error rate is less likely to reflect the effect. Conversely, nominal data contains important information on participants’ performance, especially when data is analyzed based on SDT [39–41]. In the current study, the performance index *d*’ at spatial condition showed a trend of correlation, yet response bias (i.e., a shift of decision criterion) did not. No correlations were found between EQ-SQ scores and SDT measures of the imitation condition. Meanwhile, we found that their response was biased toward the incongruent responses by analyzing SDT measures. It was indicated that the participants were more prepared for incongruent responses, which required more completed information processing [28, 33, 50, 51]. In this study, we could not find any evidence of a relationship between personal cognitive traits and an individual’s task sensitivity or response bias in the imitation condition. However, the use of SDT measures may still provide deeper insights for behavioral data in future research, such as the significant response bias found in the current study. In addition, task sensitivity in the spatial condition showed a marginal negative correlation with EQ score. This suggests poorer task sensitivity in participants who have a higher tendency toward empathic cognitions. Although the correlation was not hypothesized and did not reach significance, a relationship may exist between the processing of spatial compatibility generated by the stimuli we used and the empathizing score.

In imitation inhibition tasks, behavioral measures can be defined as the final output of information processing. Thereby, we focused on EEG mu power suppression related to the observation of action of others. By measuring in-brain action mirroring through the widely used mu ERD [17, 52, 53], we assessed the congruency effect at a lower level of information processing. As a result, there was a significant positive correlation found between Δmu ERD and SQ scores. However, no correlation between Δmu ERD and EQ was found. This suggests that participants with higher SQ scores were able to modulate mu suppression during incongruent trials, while individuals with lower SQ scores showed greater MNS activity in incongruent, compared to congruent, trials. Thus, task strategy may differ between individuals with lower and higher SQ scores; however, further study of this possibility is required. There were no correlations found in spatial condition. This suggests that the congruency effect observed in the imitation condition was likely originated from the process of automatic imitation activated by the presence of biological motion in the stimuli, not from the actual finger movement.

Notably, there were no significant correlations found for Δalpha ERD, indicating that personal traits were only correlated with congruency effects observed in sensory motor mu rhythms and not in the occipital alpha rhythm. Moreover, no significant correlation was found for the spatial condition. While the required response was exactly the same among conditions, absence of the biological motion was the factor controlled between the two conditions. This result implies that not the ability of handling spatial compatibility, but rather the ability of dealing with biological incompatibility, is correlated with the personal cognitive traits measured by the EQ-SQ questionnaire.

Measures of Δmu ERD showed significant correlations with SQ scores, while behavioral measures showed significant correlations with EQ scores. Although EQ and SQ scores are known to be moderately correlated, each score is designed to represent an independent aspect of cognition [35, 36, 54]. In addition, in the current study, no correlation was found between the two scores. As was discussed earlier, if it is assumed that a behavioral measure is the final outcome of information processing flow for the inhibition task, the mirroring activity and its inhibitory control would be placed at relatively earlier steps. Therefore, the behavioral and physiological indices may reflect different levels of processing for the inhibition of automatic imitation. According to the associative sequence learning model, automatic imitation is mediated by low-level mechanisms [14, 55, 56]. While behavioral measures reflect the whole process, the congruency effect on ERDs (i.e., ΔERDs) may reflect not only the level of imitation inhibition facilitated by higher order brain function, but also the lower level processing of observed action. In other words, not only empathic cognitive function, but also the systemizing aspect of the cognitive ability of social cognition plays an important role in successful task execution. A number of studies using fMRI to determine the brain network responsible for the inhibition of automatic imitation have been conducted [25, 26, 33, 51]. Combining these methods and personal trait data may provide deeper insight into the topic.

Because of their simplicity and popularity in the scientific field, we used EQ-SQ scores to assess cognitive traits. However, further study with many different questionnaires, combined with appropriate statistics, may provide a more robust result. Further, despite mu rhythm suppression being a widely used measure of MNS activity, there are several concerns about its quality. The major claim concerns contamination of alpha wave attenuation related to attention and visual input [21, 23]. Although simultaneous measurement of fMRI and EEG have suggested that mu rhythm is a reliable index of MNS activity, additional study with different methods to assess brain activity is required.

## Conclusions

In conclusion, this study indicated that behavioral and physiological measures of an imitation inhibition task are differentially related to personal cognitive traits. The congruency effect on the reaction time obtained from the imitation inhibition task was negatively correlated with EQ scores, and the physiological index was positively correlated with SQ scores. The application of SDT to the imitation-inhibition paradigm provided a deeper understanding to this field of study. Therefore, the current study indicated that variation in cognition is related to the inhibition of congruency effect produced by finger movements. In addition, it is suggested that the behavioral indices and the ΔERD reflect different steps of information processing. Further research with different empathizing-systemizing assessment methods and neurophysiological measures are required in the future.

## Abbreviations

ANOVA: Analysis of variances
EEG: Electroencephalogram
EQ: Empathizing quotient
ERD: Event-related desynchronization
fMRI: Functional magnetic resonance imaging
LC: Left central
MNS: Mirror neuron system
MO: Mid occipital
ROI: Region of interest
SDT: Signal detection theory
SQ: Systemizing quotient
